# Via Performance to Degradation: Oxidation Mechanism of Biodegradable Polyalphaolefin Base Oil for Electric Drive Systems

**DOI:** 10.1002/cplu.202500413

**Published:** 2025-10-23

**Authors:** Elisabeth Distler, Joachim Albrecht, Kevin Holderied, Didem Cansu Güney, Markus Kiefel, Katharina Weber

**Affiliations:** ^1^ Research Institute for Innovative Surfaces FINO Aalen University Beethovenstr. 1 D‐73430 Aalen Germany

**Keywords:** degradation, electric drive systems, oxidation, polyalphaolefins, sustainable lubricants

## Abstract

An additive‐free, low‐viscosity polyalphaolefin (PAO) has been oxidized under pure oxygen at elevated pressure and temperature. This biodegradable PAO base oil is a promising candidate for use as a motor‐gear lubricant in electrical drive systems. The oxidation behavior is systematically investigated to evaluate its thermal stability and long‐term performance. Rheological measurements are performed to assess viscosity, water content is quantified, tribological tests determine the coefficient of friction, and Fourier‐transform infrared spectroscopy is used to monitor chemical changes during oxidation. All analytical methods consistently revealed a two‐step oxidative degradation process. It is proposed that the first stage involves the formation of carbonyl compounds and water without compromising lubrication properties, while the second stage—triggered by hydrolysis of oxidation products—leads to chain scission and initiates the desired degradation. This two‐stage mechanism is discussed in the context of technological functionality and sustainability requirements for next‐generation electric drive lubricants.

## Graphical abstract

Oxidation of biodegradable polyalphaolefin for motor‐gear units in e‐drive systems. Viscosity, water content, tribological tests, and Fourier‐transform infrared spectroscopy are performed. All analytical methods show a two‐step oxidative process. In the first stage, carbonyl compounds and water are formed without compromising lubrication properties, while the second stage leads to chain scission and initiates the desired degradation.

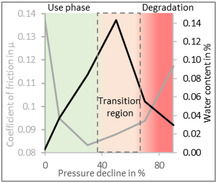

## 1 Introduction

Sustainability is one of the defining challenges of our time, with increasing emphasis on electrification in the mobility sector.^[^ ^1^ ^–^ ^3^ ^]^ Electric vehicles (EVs), as a cornerstone of this transition, impose new demands on lubricant performance.^[^ ^4^ ^]^ The integration of the electric motor and gearbox into a single housing offers potential for resource savings but introduces complex requirements for lubricants.^[^ ^5^ ^]^ In EV motor‐gear units, lubricants must simultaneously cool electric motors and lubricate drivetrains, often at rotational speeds exceeding 20,000 revolutions per minute in an operating condition of sustained excursions at high temperatures of 100 °C and above.^[^ ^6^ ^–^ ^9^ ^]^ Efficient thermal management is therefore essential.^[^ ^6^ ^,^ ^10^ ^]^ Such lubricants must be electrically nonconductive, compatible with sensitive components like copper and elastomers, and capable of rapid oil circulation—necessitating low viscosity without compromising wear protection.^[^ ^4^ ^,^ ^7^ ^,^ ^11^ ^,^ ^12^ ^]^ Moreover, recent studies have shown that lubricant composition can significantly influence critical failure mechanisms such as white etching cracks (WEC) in rolling bearings under boundary lubrication conditions^[^ ^13^ ^,^ ^14^ ^]^ as well as foaming tendencies.^[^ ^15^ ^]^ Given that ≈20% of global energy consumption is lost to friction,^[^ ^16^ ^]^ the development of high‐performance lubricants plays a crucial role in improving overall energy efficiency. From a sustainability perspective, modern lubricants are increasingly expected to degrade naturally after use to reduce environmental impact.^[^ ^2^ ^,^ ^3^ ^,^ ^17^, ^18^, ^19^ ^–^ ^20^ ^]^ However, these fluids must also maintain high functional stability under demanding operating conditions.^[^ ^21^ ^]^ This dual requirement—for long‐term performance during service life and reliable biodegradability postuse—poses a fundamental challenge in lubricant formulation and underscores the need to understand degradation mechanisms, particularly oxidative processes.^[^ ^22^ ^]^ Lubricants typically consist of base oils and performance‐enhancing additives. Among sustainability criteria such as renewability, biodegradability, and recyclability, the base oil is the most critical component.^[^ ^23^ ^,^ ^24^ ^]^ A high‐quality base oil can reduce the dependency on additives, thereby enhancing both performance and sustainability. To extend service intervals and minimize waste, synthetic and semisynthetic base oils, especially those with superior oxidation resistance, are increasingly favored.^[^ ^16^ ^,^ ^25^ ^]^ Polyalphaolefins (PAOs), synthetic base oils produced via oligomerization of alpha‐olefins,^[^ ^26^ ^]^ are among the most promising candidates due to their thermal and oxidative stability, favorable lubrication characteristics, and potential for biodegradability.^[^ ^27^ ^,^ ^28^ ^]^ To better understand oxidative degradation and its impact on lubricant properties, this study employs a model PAO system with a biodegradability of 71% according to Organization for Economic Co‐operation and Development (OECD) 301B. It is free of stabilizing additives and intrinsic oxygen. This enables a controlled investigation of oxidation dynamics and underlying degradation mechanisms. A clear understanding of oxidative effects not only aids in lubricant design but also supports condition‐based maintenance strategies, further contributing to environmental sustainability by extending service intervals and reducing premature oil disposal.^[^ ^29^ ^]^ The impact of oxidation on key oil properties like viscosity, coefficient of friction (COF), water content, and chemical structure were investigated.

## 2 Experimental Section

**Figure 1:**
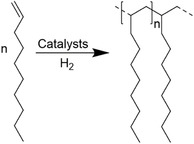
Synthesis of polyalphaolefins based on dec‐1‐ene as the monomeric basis.^[^ ^30^ ^]^

PAOs are saturated oligomers produced through the catalytic oligomerization of alpha‐olefins, with most processes using 1‐decene as the monomer (**Figure** **1**).^[^ ^30^ ^]^ At low viscosities, PAOs mainly consist of dimers, the majority of which contain isomeric side‐chain structures.^[^ ^31^ ^–^ ^33^ ^]^

In the initial step of PAO synthesis, olefins undergo oligomerization using various acidic catalysts, resulting in oligomers with different molecular weights. The second step involves hydrogenating the unsaturated oligomers to render them chemically inert and enhance their stability.^[^ ^34^ ^]^ The increasing number of available catalytic processes allows for the production of customized PAOs. A key advantage of this is the ability to control the final product's properties, such as viscosity and viscosity index.^[^ ^30^ ^]^ Furthermore, short‐chained PAOs can achieve rather high OECD levels of biodegradability.^[^ ^35^ ^]^ The oil used in this study is a synthetic, nonpolar PAO base oil (Zeller‐Gmelin, Eislingen/Fils, Germany), consisting of dimers of 1‐decene. In this study, the base oil was tested without additives. It has a low kinematic viscosity *ν* of 5 mm^2^ s^–1^ at 40 °C and 2 mm^2^ s^–1^ at 100 °C. The specific gravity at 15 °C is 0.8 g cm^–3^.

The PAO was oxidized using a Rapid Oxy 100 oxidation tester (Anton Paar GmbH) under controlled conditions in accordance with ASTM D8206 to investigate its aging behavior. The oxidation process was carried out at two temperatures of 120 or 140 °C, with an initial pressure of 7 bar, using pure oxygen as the oxidation medium. The progression of oxidation was monitored by defining the stop criterion as a pressure drop between 10% and 90% of the highest measured pressure (*p*_max_). Data acquisition was performed continuously at 1 s intervals, ensuring precise tracking of pressure changes throughout the oxidation process.

The formation of oxidation products was monitored using ultraviolet–visible (UV/vis) spectroscopy. Measurements were carried out with an Agilent Cary 60 spectrophotometer using a quartz cuvette with a 1 mm path length. To ensure consistent optical conditions and avoid concentration‐dependent absorbance effects, PAO samples were diluted with *n*‐heptane prior to UV/vis measurement. For all diluted samples, the absorbance spectrum of pure *n*‐heptane was recorded and subtracted as a blank from the raw data. In selected cases, highly oxidized samples were also measured in their undiluted state to capture absorbance changes in the visible range. In these instances, no blank subtraction was applied.

The rheological behavior of the PAO samples was characterized to evaluate the impact of oxidation on viscosity. Measurements were carried out using a rotational rheometer (Anton Paar MCR 102e) equipped with a cone‐plate geometry (75 mm diameter, 1° cone angle). All tests were performed at room temperature, with a fixed measurement gap of 0.15 mm and a sample volume of 2300 µL. The dynamic viscosity was recorded while linearly increasing the shear rate from 0.1 to 1000 s^−1^ over a total measurement time of 205 s.

The water content of fresh and oxidized PAO samples was determined by volumetric Karl Fischer titration to assess the influence of oxidation on water accumulation. Measurements were performed using a Metrohm Titrando 852 titration system with a methanol‐based Karl Fischer reagent.

The tribological properties of fresh and oxidatively aged PAO samples were evaluated using an easy tribology screener (ETS) tribometer (Optimol Instruments) in accordance with DIN 51834. The contact pair consisted of a polished disc (base body) and a spherical counter body, both made of hardened 100Cr6 steel (62 HRC). The counter body was subjected to sinusoidal translational motion across the stationary disc. A 150 µL volume of PAO was applied between the friction partners. Tests were conducted for 2 h under a normal load of 250 N, at a frequency of 50 Hz, with a stroke length of 1 mm at room temperature. This setup enabled the assessment of oxidation effects on the COF.

Fourier‐transform infrared (FTIR) spectroscopy was employed to monitor chemical changes in the fresh and oxidized PAO samples. Spectra were recorded using a Bruker Alpha II spectrometer equipped with a diamond attenuated total reflectance (ATR) crystal. Measurements were performed over the spectral range of 4000–400 cm^−1^, averaging 24 scans per sample to ensure reproducibility. Baseline correction was applied to all spectra to allow for direct comparison.

## 3 Results

### 3.1 Oxidation

**Figure 2:**
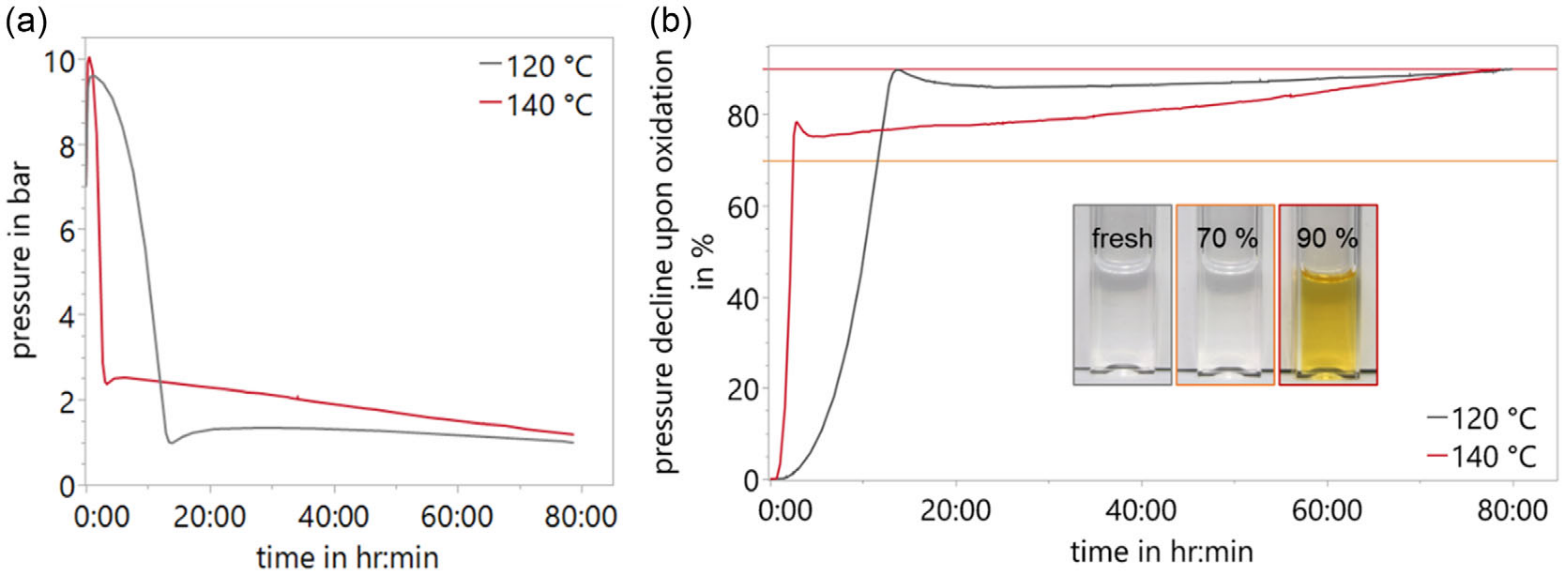
Oxidation of polyalphaolefin base oil. a) Pressure declines of oxygen in bar in pressure chamber with 5 ml PAO base oil at 120 and 140 °C. b) Pressure declines of oxygen in % at 120 and 140 °C. Representative pictures show that fresh base oil is clear, after 70% pressure decline upon oxidation the oil is still transparent, while it visibly changes its color to yellow after 90% pressure decline upon oxidation.

Since oxidation rates are highly temperature‐dependent, the chosen temperatures reflect thermal conditions encountered in motor‐gear units.^[^ ^8^ ^,^ ^9^ ^]^ The oxidation process was monitored using a sealed chamber and recording the oxygen pressure over time. **Figure** **2**a shows the pressure–time profiles during oxidation at two temperatures (120 and 140 °C) over a period of three days. Oxidation was initiated at an oxygen pressure of 7 bar at room temperature. As the temperature increased, the pressure rose due to gas expansion, reaching a maximum of ≈10 bar. Upon reaching this peak pressure (*p*_max_), oxygen consumption commenced, as evidenced by a subsequent pressure decline. At 140 °C (red curve), the pressure decreases rapidly to ≈2 bar. This is followed by a slower decline until the predetermined endpoint, defined as 90% pressure decline upon oxidation, at 1 bar. At 120 °C (gray curve), the pressure decreases fast to ≈1 bar; however, this decline is slower than that observed at 140 °C. The pressure then stays close to 1 bar of oxygen pressure until it finally reaches its stop criterion of 90% pressure decline upon oxidation at slightly less than 1 bar. The representation of Figure 2b illustrates the same data as percentage pressure decline versus time. The percentage of pressure decline upon oxidation is rising rapidly up until 80% at 140 °C (red curve), resp. almost 90% at 120 °C (gray curve). The increase is slower afterward until the predetermined endpoint of 90% pressure decline upon oxidation is reached. The two parts of different slopes are separated by a transient overshoot of the pressure that is originated by the release of physically dissolved oxygen. For the considered oxidation and degradation process of the base oil, this effect is negligible. Photos of the oil at particular stages of the process are added to the Figure. A clear transition from transparent to yellow is found toward the end of the process. The oxidation process observed in Figure 2 consists of two stages, as evidenced by both the pressure decay curve and the base oil's color change: 1) Initial rapid pressure drop: This phase is marked by a sharp decline in pressure immediately after reaching *p*_max_, with an ≈80% reduction occurring within a relatively short timeframe. During this stage, oxygen is either dissolved in the PAO base oil or incorporated into its molecular structure. At 140 °C, the steeper pressure drop indicates a higher oxidation rate compared to 120 °C. These findings underscore the temperature dependence of PAO's oxidative stability. Higher temperatures accelerate oxidation and oxygen consumption, particularly in this initial phase. Notably, no visible color change occurs during this stage. 2) Gradual pressure decline: In this phase, the more gradual decline in pressure indicates a slower oxidation rate. The pressure decline continues until the stop criterion is reached, defined as a 90% reduction from *p*_max_. Oxygen consumption slows significantly in this stage, and a visible color change occurs when the pressure decline falls between 70% and 90% pressure decline, indicating the formation of additional chemical species.

### 3.2 UV/Vis Spectroscopy

**Figure 3:**
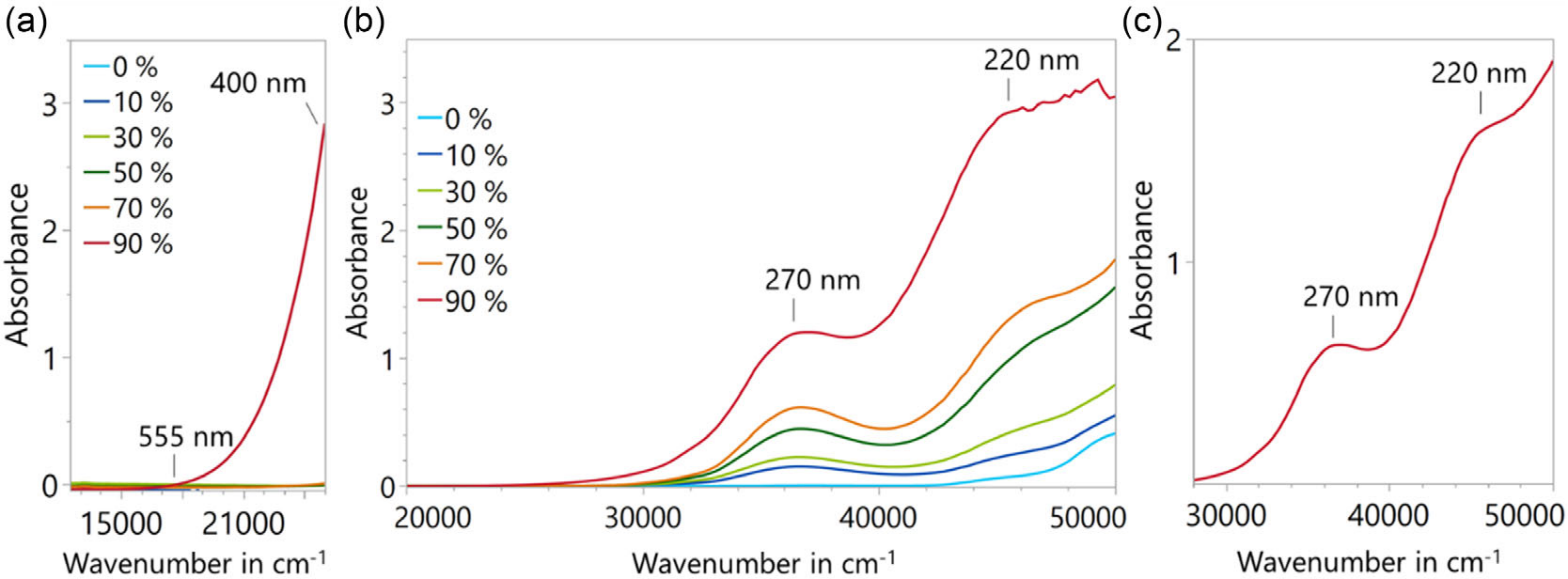
UV/vis spectra of non‐additivated polyalphaolefin a) undiluted at different oxidation levels, b) diluted 1:10 in *n*‐heptane, and c) at 90% pressure decline upon oxidation diluted 1:20 in *n*‐heptane.

The UV/vis spectroscopy results shown in **Figure** **3**a display the absorbance spectra of undiluted PAO samples that were aged to different levels of oxidation, as indicated by pressure decline. Only the sample with 90% pressure decline exhibits significant absorbance in the visible region between 18,000 cm^−1^ (555 nm) and 25,000 cm^−1^ (400 nm), giving rise to visible yellowing depicted in Figure 2. Figure 3b shows spectra of the PAO samples diluted 1:10 in *n*‐heptane to avoid concentration‐dependent absorbance effects in the UV region. A progressive rise in absorbance with increasing pressure decline upon oxidation is observed at 37,000 cm^−1^ (270 nm), corresponding to n–π* transitions in carbonyl (C=O) groups. The intensity of this peak increases steadily from 0 at 0 % pressure decline to 0.6 at 70 % pressure decline, and more than doubles at 90% pressure decline, reaching a maximum of 1.3. At 45,500 cm^−1^ (220 nm), attributed to π–π* transitions in C=O groups, a similar trend is observed. The absorbance increases from 0.05 at 0% pressure decline to 1.2 at 70% pressure decline and further to ≈3.0 at 90% pressure decline upon oxidation. Due to the high absorbance values observed at 90% pressure decline upon oxidation, this sample was further diluted 1:20 in *n*‐heptane. The corresponding spectrum is depicted in Figure 3c. At 37,000 cm^−1^ (270 nm), the absorbance is ≈0.5 and at 45,500 cm^−1^ (220 nm), it reaches ≈1.6.

### 3.3 Rheology

**Figure 4:**
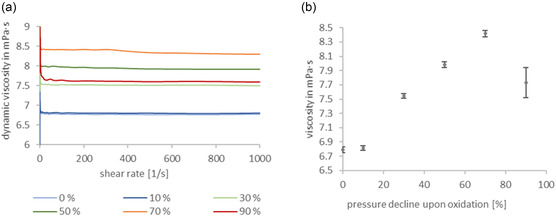
Rheological behavior of non‐additivated polyalphaolefin at different pressure decline upon oxidation in %. a) Raw data of rheological experiments, showing a shear rate independent viscosity. b) Mean values of dynamic viscosity values of polyalphaolefin base oil at different pressure declines upon oxidation.

**Figure** **4**a presents the dynamic viscosity of PAO base oil samples with varying degrees of pressure decline upon oxidation as a function of shear rate, ranging from 0.1 to 1000 s^−1^. For all samples, the viscosity remains constant beyond a shear rate of 100 s^−1^, indicating shear rate‐independent behavior at higher shear rates. Given the observed shear rate independence above 100 s^−1^, mean dynamic viscosity values were calculated across this range for each sample. These averaged values, which help to minimize measurement noise and minor variations, are displayed in Figure 4b, providing representative viscosity values for each level of pressure decline. The data reveal that with increasing pressure decline upon oxidation, the viscosity initially rises from ≈6.8 mPa·s in the unaged sample to around 8.4 mPa·s at 70% pressure decline. Further oxidation results in a decrease in viscosity, reaching ≈7.7 mPa·s at a 90% pressure decline. The initial rise likely results from enhanced intermolecular interactions of oxidation products with intact oligomeric structure, while further oxidation leads to breakdown into lower‐viscosity compounds. This suggests a two‐step process: oxidation‐induced intermolecular interactions involving hydrogen‐bonding through carbonyl groups initially increase viscosity, while thermal‐oxidative cleavage of C—C chains reduces it at higher oxidation levels.

### 3.4 Water Content

**Figure 5:**
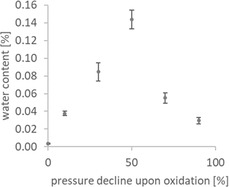
Water content of polyalphaolefin base oil at different pressure declines upon oxidation.

**Figure** **5** presents the water content of PAO samples with increasing pressure decline upon oxidation. The graph shows an initial increase in water content from close to 0% in the fresh oil, peaking at ≈0.14% at 50% pressure decline upon oxidation. This is followed by a subsequent decrease to ≈0.03% at 90% pressure decline. This behavior suggests that first water accumulates during the oxidation process, probably as a byproduct of a radical aging mechanism, but is reduced at higher oxidation levels due to subsequent chemical reactions, like a nucleophilic mechanism leading to chain scission.

### 3.5 Tribology

**Figure 6:**
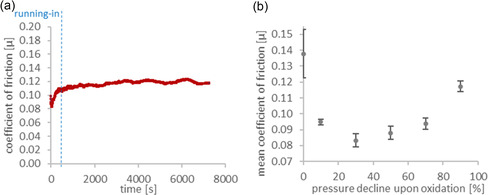
Tribological measurements of polyalphaolefin base oil. a) Raw data of the coefficient of friction of a PAO sample oxidized until 90% pressure decline upon oxidation and b) Mean values of the coefficient of friction of different PAO samples (0, 10, 30, 50, 70, and 90% pressure declines upon oxidation).

During tribological measurements, the coefficient of friction (COF) was determined for the specific tribological contact using PAO base oil as the lubricant. **Figure** **6**a shows the tribological measurement curve of the COF of the most oxidized sample (90% pressure decline upon oxidation) over time. Apart from the running‐in region up to ≈250 s, a mainly constant COF of about *µ* = 0.12 is found. The mean COF value from this constant phase is used for further analysis, as it reflects the steady‐state behavior of the system.

Figure 6b shows the mean values of the COF for PAO samples at different pressure declines upon oxidation. Initially, the fresh oil exhibits the highest COF, around *µ* = 0.15. As oxidation progresses to a 10% pressure decline, the COF decreases significantly. The lowest COF is observed between 30% and 50% pressure decline, where it stabilizes around *µ* = 0.09–0.10. Beyond 50%, the COF gradually increases again, reaching ≈*µ* = 0.09 at 70% pressure decline and rising to around *µ* = 0.12 at 90% pressure decline upon oxidation. This non‐monotonous behavior is observed and attributed to two individual processes of oil oxidation. The first process reduces friction and is dominant at low oxidation values. The second process, prevalent at higher oxidation levels, leads to an increase in friction. This aligns with the two‐step interpolation observed in oxidation and rheology measurements.

### 3.6 FTIR Spectroscopy

**Figure 7:**
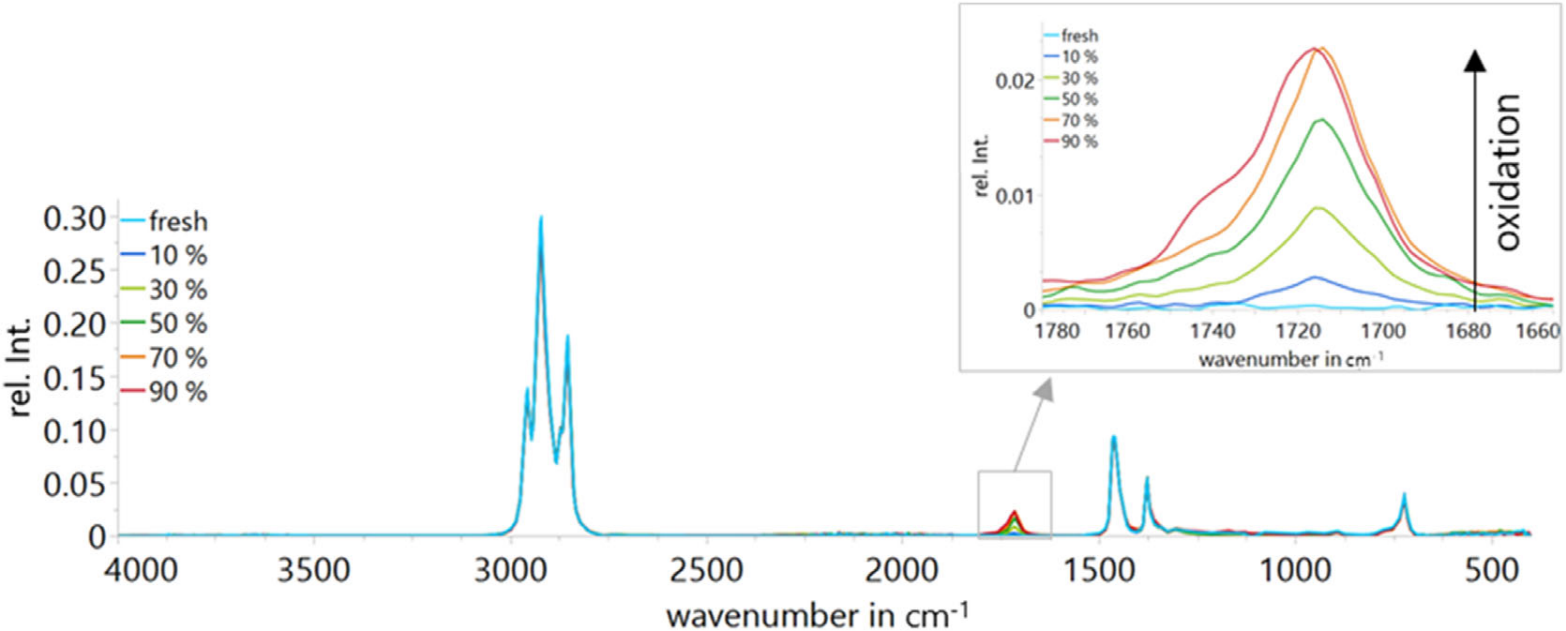
Baseline corrected FTIR spectra of polalphaolefin base oil at different pressure declines upon oxidation in %.

To elucidate the chemical mechanisms occurring during oxidation, FTIR measurements were performed on PAO samples at different stages of oxidative aging, indicated by pressure decline. The resulting spectra are presented in **Figure** **7**. These spectra provide insight into the chemical changes that occur during the oxidation of PAO under controlled conditions. In the fingerprint region (400–1600 cm^−1^), subtle but noticeable changes appear, indicating structural modifications of the base oil. In particular, slight increases in absorption bands corresponding to C—O and C—C vibrations suggest the formation of oxygen‐containing species such as alcohols and esters. The inset of Figure 7 shows the magnified region from 1650 to 1850 cm^−1^. In the light blue curve, representing the fresh oil, no distinct bands are observed across the spectrum. As the oxidation level increases, a band begins to emerge around 1715 cm^−1^ and grows monotonously up until 70% pressure decline upon oxidation. From ≈30% pressure decline upon oxidation, an additional increase appears in the region between 1800 and 1740 cm^−1^. Between 70% and 90% pressure decline, the intensity at 1715 cm^−1^ no longer increases significantly; however, a clearly visible shoulder develops around 1741 cm^−1^.

**Figure 8:**
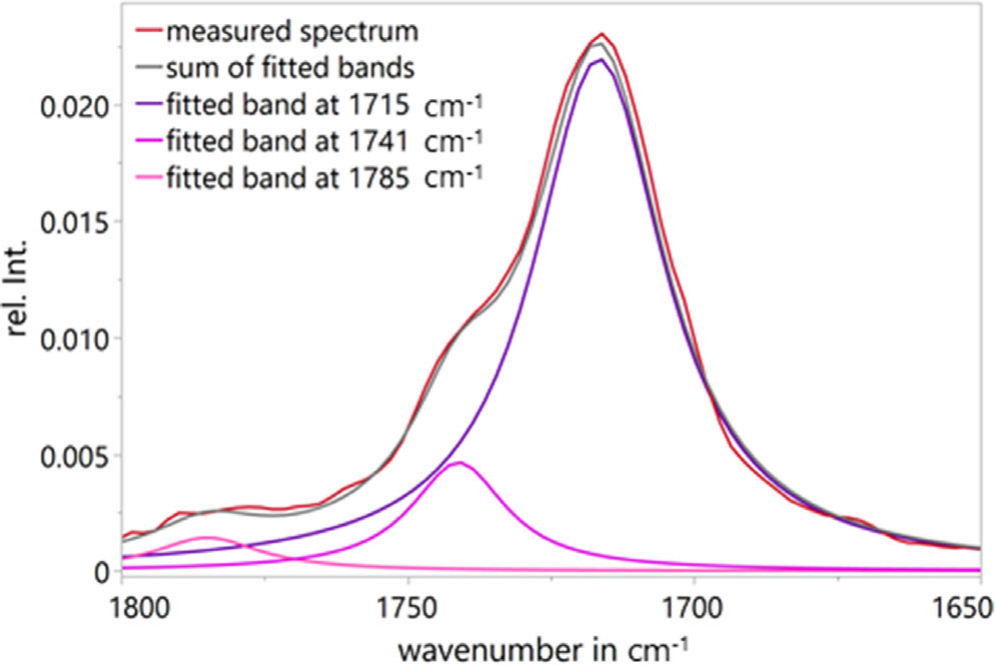
Measured FTIR spectrum (red) and fitted bands at 1715 cm^−1^ (dark purple), 1741 cm^−1^ (light purple), and 1785 cm^−1^ (pink) of 90% pressure decline PAO base oil. The gray line is the sum of the three fitted bands.

To gain deeper insight into the oxidation process, the carbonyl absorption region was fitted using Lorentzian peak fitting at each local maximum according to Levenberg–Marquardt algorithm. The result of the curve at 90% pressure decline upon oxidation is depicted in **Figure** **8**. The fitted band at 1715 cm^−1^ is attributed to the C=O stretching vibration of a carbonyl group from a ketone.^[^ ^36^ ^,^ ^37^ ^]^ This band is relatively broad and features a shoulder at 1741 cm^−1^, which suggests the presence of additional carbonyl‐containing species, such as carboxylic esters (fitted band at 1741 cm^−1^).^[^ ^36^ ^,^ ^37^ ^]^ Additionally, a lower band at 1785 cm^−1^ presumably corresponds to the C=O stretching of a non‐hydrogen‐bonded carboxylic acid.^[^ ^36^ ^,^ ^37^ ^]^ This is consistent with the previously described decline of the water content at higher oxidation levels. At this stage of oxidation, water molecules are consumed in nucleophilic reactions on carboxylic species and are less available as a hydrogen‐bonding partner for the formation of carboxylic acids. Since the absorption band at 1785 cm^−1^ is much smaller than the other bands, we focus in the following section on the bands at 1715 and 1741 cm^−1^.

**Figure 9:**
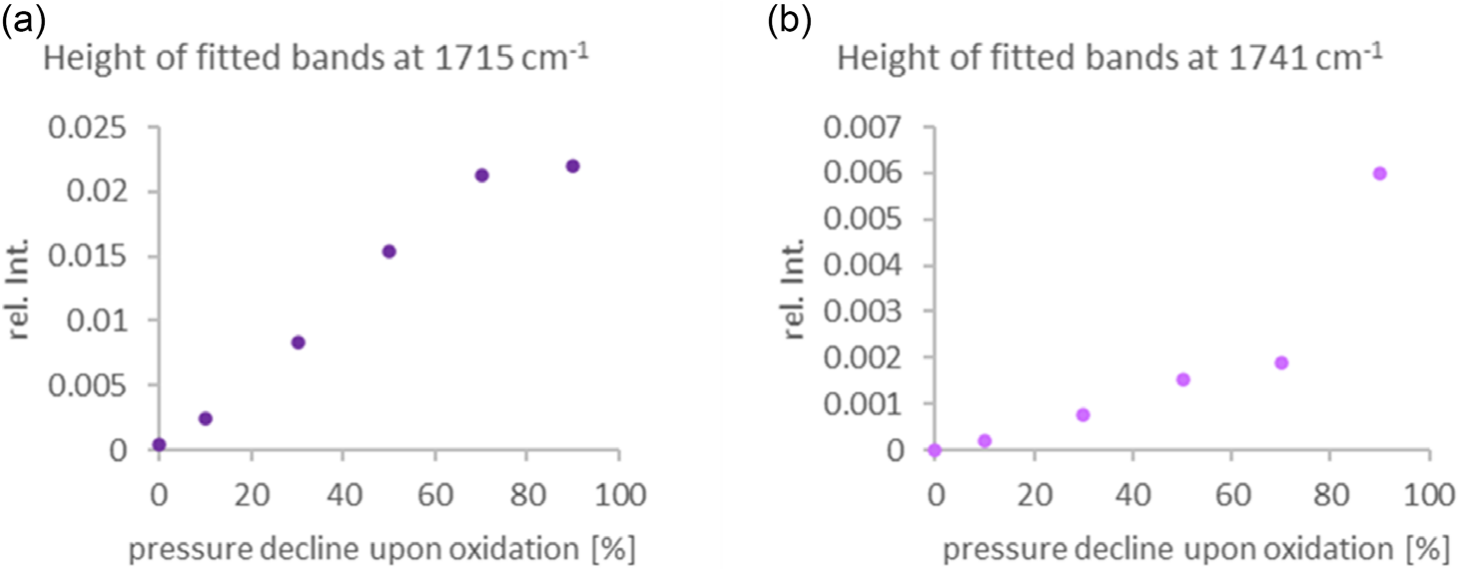
Height of fitted bands of FTIR spectrum at different pressure declines upon oxidation a) at 1715 cm^−1^; b) at 1741 cm^−1^.

The intensities of the fitted bands at 1741 and 1715 cm^−1^ across various pressure declines upon oxidation are presented in two separate plots in **Figure** **9**. In Figure 9 the data for 1715 cm^−1^ band display a steady increase in relative intensity, starting near zero in the fresh oil. This upward trend continues with progressing pressure decline and levels off around 70% pressure decline upon oxidation. This suggests that the formation of carbonyl‐related species associated with this band largely occurs in the early to mid‐stages of the oxidation process. The change in intensity between 70% and 90% is minimal, implying a saturation in the formation of these species.

In contrast, Figure 9b displays the intensity profile of the 1741 cm^−1^ band, which differs notably. While a gradual increase is observed up to 70% pressure decline, a marked rise in intensity occurs between 70% and 90% pressure decline upon oxidation, increasing from –0.006. This more pronounced late‐stage increase, compared to the 1715 cm^−1^ band, indicates a different formation dynamic—likely reflecting the emergence of distinct oxidation products that become more prominent in the advanced stages of degradation.

**Figure 10:**
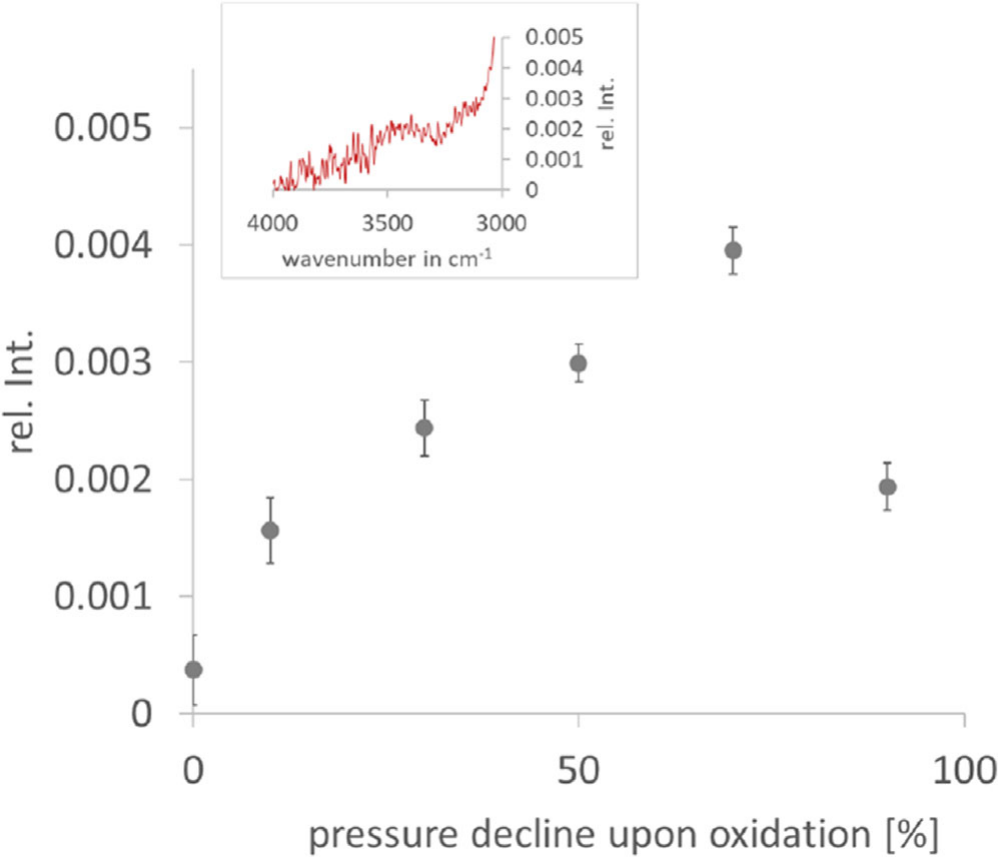
Height of the FTIR absorption band at 3400 cm^−1^ for PAO at varying degrees of pressure decline upon oxidation. The inset displays the raw spectral data corresponding to a 90% pressure decline upon oxidation, recorded in the 4000–3000 cm^−1^ region.

The raw FTIR spectral data in the range of 4000–3000 cm^−1^, presented in **Figure** **10**, indicate an increasing trend in absorption with progressive pressure decline upon oxidation up to 70% in the O—H stretching absorption band (3600–3230 cm^−1^).^[^ ^38^ ^]^ Interestingly, the spectrum corresponding to a 90% pressure decline exhibits a lower intensity and aligns more closely with those of moderately oxidized samples, specifically within the range of 10% to 30% pressure decline upon oxidation. The band is broadened due to hydrogen bonding.^[^ ^38^ ^]^

## 4 Discussion

Our investigations target the development of a biodegradable lubricant for motor‐gear units in electric drive systems, with a focus on fulfilling a two‐stage performance requirement: maintaining functional stability throughout the intended service life, followed by controlled degradation to ensure environmental compatibility after end‐of‐life. Our results indicate the presence of two distinct oxidative processes of PAO at 140 °C and an initial oxygen pressure of 7 bar: an initial fast oxidation reaction followed by a slower degradation process. This biphasic behavior is evident in the oxygen pressure drop over time that reflects the extend of oxidation. ≈80% of the total oxygen uptake occurs during the first phase, marked by a sharp pressure drop. Compared to the first, the second process shows a significantly slower reduction in oxygen pressure. Multistage oxidation processes in hydrocarbons have been reported in the literature,^[^ ^39^ ^]^ highlighting the presence of distinct reaction stages with varying chemical mechanisms.

Upon oxidation, the sample exhibited a yellow coloration at 90% pressure decline. UV/vis spectroscopy displays absorbance between 400 and 550 nm in these highly oxidized samples. Furthermore, it supports the presence of oxidation products in all oxidized samples, with absorbance increases at 220 and 270 nm corresponding to π–π* and n–π* transitions in carbonyl (C=O) groups. Notably, these absorbance signals increase more sharply for the most highly oxidized samples, indicating an accelerated formation of carbonyl‐containing degradation products.

**Figure 11:**
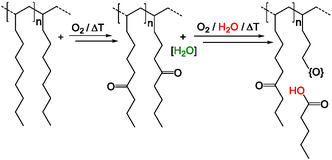
Proposed two‐step oxidative degradation mechanism of polyalphaolefin‐based lubricants. In the first step, oxidation under elevated temperature and oxygen atmosphere leads to the formation of ketone groups at the side chains via radical pathways, accompanied by the formation of water. In the second step, under continued oxidative conditions and in the presence of water as a nucleophile, further reactions occur, leading to chain scission and the formation of carboxylic acids.^[^ ^39^ ^]^

Rheological analyses revealed a distinct increase in dynamic viscosity during the initial oxidation process. This is attributed to the formation of polar oxidation products, such as ketones, likely through a radical oxidation mechanism (**Figure** **11**). These polar compounds promote intermolecular interactions, increasing viscosity and thereby slowing further radical propagation. This leads to a self‐limiting mechanism, where radicals become effectively trapped within the increasingly viscous medium.^[^ ^39^ ^]^ At more advanced stages of oxidation, this trend reverses: viscosity decreases, indicating molecular degradation via chain scission. This transition is supported by water content measurements that show an initial increase—likely due to water formation as a byproduct of radical oxidation—followed by a systematic decline beyond ≈50% pressure decline upon oxidation. This points to the onset of a water‐consuming reaction mechanism, likely hydrolysis. The ketone‐containing intermediates are converted into carboxylic acids, accompanied by chain scission and structural degradation of the base oil. We propose that this marks the transition to a nucleophilic pathway, in which water plays a central role in further molecular breakdown. As long as water remains available, the nucleophilic pathway dominates, suggesting that viscosity is a critical control parameter: it governs the mobility of water and radicals, thereby dictating the transition from the stabilizing first process to the degradative second process. In this context, viscosity is not only a mechanistic indicator but also a key performance metric. Infrared (IR) spectroscopy confirms these chemical transformations, showing the formation of specific functional groups characteristic of each oxidation process. First, the band at 1715 cm^−1^, which is attributed to the C=O stretching vibration of a carbonyl group from a ketone,^[^ ^31^ ^,^ ^32^ ^]^ rises with increasing pressure decline upon oxidation. At high oxidation levels, a shoulder at 1741 cm^−1^ suggests the presence of additional carbonyl‐containing species, such as carboxylic esters.^[^ ^31^ ^,^ ^32^ ^]^ Additionally, a lower band at 1785 cm^−1^ presumably corresponds to the C=O stretching of a non‐hydrogen‐bonded carboxylic acid.^[^ ^31^ ^,^ ^32^ ^]^

**Figure 12:**
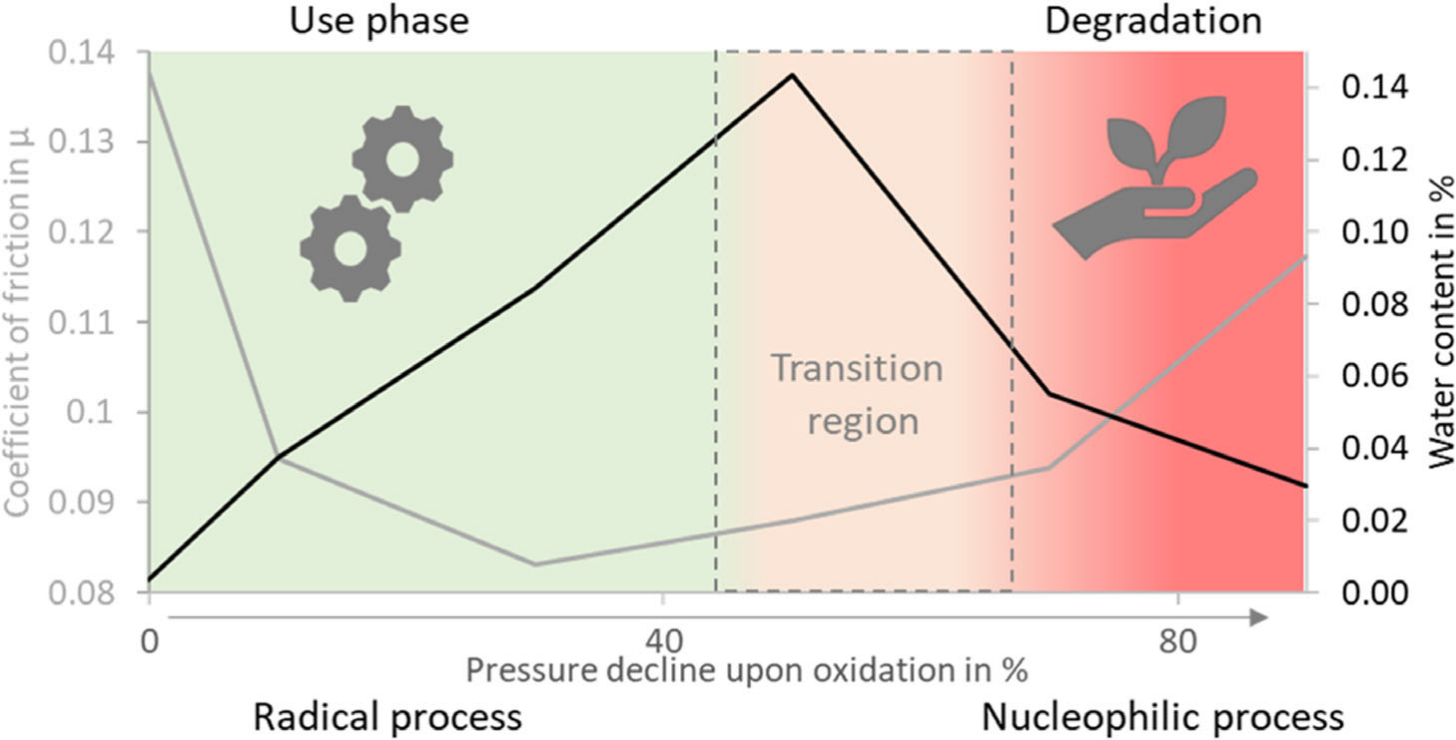
Coefficient of friction (gray) and water content (black) with respect to the level of oxidation. We propose a radical mechanism in low oxidation levels (use phase) and a nucleophilic process at high oxidation levels (degradation phase).

In practical terms, this biphasic oxidation behavior is reflected in the tribological performance. The COF initially decreases, likely due to the increased viscosity and the formation of a thicker lubricant film. However, with continued oxidation, the COF gradually rises in moderately oxidized samples and increases significantly in highly oxidized ones—correlating with molecular breakdown and loss of lubricating functionality. These findings suggest that moderate oxidation during the first process may not impair, and may even enhance, lubricant performance—a potentially advantageous feature for applications such as high‐speed electric drivetrains. However, once the system transitions into the second oxidative phase, performance and structural integrity decline rapidly. **Figure** **12** illustrates the correlation between the COF and the water content throughout the oxidation process.

Conventional antioxidants, such as hindered phenols and aromatic amines, operate primarily by scavenging free radicals, thus suppressing or delaying the onset of the first radical oxidation mechanism.^[^ ^40^ ^,^ ^41^ ^]^ This also delays the progression into the more damaging second process. Alternatively, additives that scavenge or bind water could selectively inhibit the hydrolytic degradation pathway, offering a complementary strategy to prolong lubricant life. The clear identification of two chemically distinct oxidative stages has important implications for lubricant formulation and condition monitoring. Understanding these mechanisms allows for the development of targeted additive systems that selectively stabilize the early oxidation mechanism, delay or suppress the destructive second process, and enable real‐time monitoring through viscosity or water content as key indicators. In contrast, biological degradation processes typically proceed via nucleophilic mechanisms.^[^ ^42^ ^]^ This raises the question of whether optimizing nucleophilic conditions—such as increased water availability or thermal activation—could accelerate or steer the biodegradation process more effectively.

## 5 Conclusion

The oxidative degradation of synthetic lubricants in electric drivetrain systems proceeds via a distinct two‐step mechanism. The initial rapid oxidation process involves the formation of polar functional groups, primarily ketones, accompanied by increased viscosity and water content. This stage does not impair, and may even temporarily enhance, performance‐relevant properties such as the COF due to increased lubricant film thickness. In contrast, the subsequent slower process is characterized by water‐consuming reactions and chain scission, leading to the formation of carboxylic acids, viscosity loss, and increased friction, signaling the onset of functional degradation. IR spectroscopy confirms these transitions at the molecular level, distinguishing chemical transformation from mere oxygen dissolution. Conventional antioxidants effectively inhibit the initial radical‐driven oxidation and can thereby delay or prevent entry into the second, more destructive phase. Alternatively, water‐scavenging additives may offer a targeted approach to suppress late‐stage degradation. Monitoring physical and chemical oil parameters could enable condition‐based maintenance strategies and extended service intervals. Finally, controlled water exposure may serve as a trigger for end‐of‐life biodegradation, aligning lubricant design with circularity and sustainability goals.

## Conflict of Interest

The authors declare no conflict of interest.

## Data Availability

The data that support the findings of this study are available from the corresponding author upon reasonable request.

## References

[1] C. Saha , in Lubricants from Renewable Feedstocks (Eds: S. Pradhan , L. Prasad , C. Madankar , S. N. Naik ), WILEY 2024, p. 1212.

[2] E. Beran , Tribol. Int. 2008, 41, 1212.

[3] M. K. Pathak , A. Joshi , K. K. S. Mer , J. K. Katiyar , K. V. Patel , Springer eBooks Chemistry and Materials Science, Springer, Singapore 2019, 197.

[4] L. I. Farfan‐Cabrera , Tribol. Int. 2019, 138, 473.

[5] J. Montonen , J. Nerg , M. Polikarpova , J. Pyrhonen , IEEE Acc. 2019, 7, 69108.

[6] L. I. Farfan‐Cabrera , A. Erdemir , Electric Vehicle Tribology: Challenges and Opportunities for a Sustainable Transportation Future, Elsevier, Amsterdam, London, Cambridge, MA 2024.

[7] T. Newcomb , Front. Mech. Eng. 2023, 9, 1139385.

[8] L. He , B. Tong , Y. Zhang , D. Guo , J. Electr. Eng. Technol. 2024, 19, 4381.

[9] R. W. Johnson , J. L. Evans , P. Jacobsen , J. Thompson , M. Christopher , IEEE Trans. Electron. Packag. Manufact. 2004, 27, 164.

[10] B. Morhard , D. Schweigert , M. Mileti , M. Sedlmair , T. Lohner , K. Stahl , Forsch. Ingenieurwes. 2021, 85, 443.

[11] T. König , L. Cadau , L. Steidle , D. C. Güney , J. Albrecht , K. Weber , M. Kley , Forsch. Ingenieurwes. 2023, 87, 1069.

[12] K. Holmberg , P. Andersson , A. Erdemir , Tribol. Int. 2012, 47, 221.

[13] W. Holweger , A. Schwedt , V. Rumpf , J. Mayer , C. Bohnert , J. Wranik , J. Spille , L. Wang , Lubricants 2022, 10, 24.

[14] J. Wranik , W. Holweger , T. Lutz , P. Albrecht , B. Reichel , L. Wang , Lubricants 2022, 10, 96.

[15] L. Hafner , J. Holzbrecher , M. Wiedenmann , T. Hieronymus , S. Schwarzer , F. Dohnal , J. Phys.: Conf. Ser 2024, 2909, 012012.

[16] K. Holmberg , A. Erdemir , Tribol. Int. 2019, 135, 389.

[17] P. Nowak , K. Kucharska , M. Kamiński , Int. J. Environ. Res. Public Health 2019, 16, 3002. 31434340 10.3390/ijerph16163002 PMC6720566

[18] J. C. Ssempebwa , D. O. Carpenter , J. Hazard. Mater. 2009, 161, 835. 18513868 10.1016/j.jhazmat.2008.04.028 PMC2643015

[19] A. Z. Syahir , N. Zulkifli , H. H. Masjuki , M. A. Kalam , A. Alabdulkarem , M. Gulzar , L. S. Khuong , M. H. Harith , J. Clean. Prod. 2017, 168, 997.

[20] P. Nagendramma , S. Kaul , Renew. Sustain. Energy Rev. 2012, 16, 764.

[21] D. C. Güney , T. König , D. Proksch , M. Kley , J. Albrecht , K. Weber , Tribol. Schmierungstech. 2025, 4, 10.24053/TuS-2024-0028.

[22] D. C. Güney , V. Joukov , J. Albrecht , K. Weber , Materialwiss. Werkstofftech 2023, 54, 1390.

[23] M. A. I. Malik , M. A. Kalam , M. A. Mujtaba , F. Almomani , Environ. Technol. Innov. 2023, 32, 103366.

[24] J. Pichler , R. Maria Eder , C. Besser , L. Pisarova , N. Dörr , M. Marchetti‐Deschmann , M. Frauscher , Green Chem. Lett. Rev. 2023, 16, 2185547.

[25] A. Tripathi , R. Vinu , Lubricants 2015, 3, 54.

[26] B. Sharma , A. Verma , A. Kukrety , S. Saini , U. Kumar , Polym. Eng. Sci. 2023, 63, 1691.

[27] G. Biresaw , Tribol. Lett. 2018, 66,

[28] Y. Chen , H. W. z. He , Y. Li , H. Wang , Lubricants 2025, 13, 62.

[29] J. M. Wakiru , L. Pintelon , P. N. Muchiri , P. K. Chemweno , Mech. Syst. Signal Proc. 2019, 118, 108.

[30] S. Ray , P. V. C. Rao , N. V. Choudary , Lubr. Sci. 2012, 24, 23.

[31] P. Panwar , E. Schweissinger , S. Maier , S. Hilf , S. Sirak , A. Martini , J. Mol. Liq. 2022, 359, 119215.

[32] A. Hanifpour , N. Bahri‐Laleh , A. Mohebbi , M. Nekoomanesh‐Haghighi , Iran Polym. J. 2022, 31, 107.

[33] S. Scheuermann , S. Eibl , P. Bartl , Lubr. Sci. 2011, 23, 221.

[34] G. D. Yadav , N. S. Doshi , Green Chem. 2002, 4, 528.

[35] J. F. Carpenter , J. Synth. Lubr. 1995, 12, 13.

[36] S. Campen , C. W. J. Fong , W. Song , J. S. Wong , Tribol. Int. 2022, 170, 107492.

[37] K. Takei , R. Takahashi , T. Noguchi , J. Phys. Chem. B. 2008, 112, 6725. 18452327 10.1021/jp801151k

[38] K. T. Sutar , P. U. Singare , Asian J. Chem. 2018, 30, 2079.

[39] K. Gerry , Ph.D. Thesis, Imperial College London (UK) 2009.

[40] M. C. Foti , J. Pharm. Pharmacol. 2007, 59, 1673. 18053330 10.1211/jpp.59.12.0010

[41] M. C. Foti , R. Amorati , G. F. Pedulli , C. Daquino , D. A. Pratt , K. U. Ingold , J. Org. Chem. 2010, 75, 4434. 20527908 10.1021/jo100491a

[42] P. J. J. Alvarez , Process Fundamentals and Mathematical Models, Wiley, Hoboken, NJ 2006.

